# Trends and disparities in liver failure-related mortality in adults with mental and behavioral disorders due to tobacco use: A retrospective analysis

**DOI:** 10.1097/MD.0000000000048719

**Published:** 2026-05-15

**Authors:** Sarim Hassan Shahab, Zuha Maryam, Syeda Malika Naqvi, Muhammad Ahmed, Alisha Matloob, Hadia Ghazala Masood, Esha Umair, Muddassir Khalid, Muhammad Talha, Ubaid Ur Rehman

**Affiliations:** a Nishtar Medical University, Multan, Pakistan; b King Edward Medical University, Lahore, Pakistan.

**Keywords:** liver failure, MBDs, mortality trends, tobacco use

## Abstract

This study aims to examine the national trends and disparities in mortality caused by liver failure among adult patients diagnosed with mental and behavioral disorders (MBDs) that are attributable to tobacco use in the United States using the Centers for Disease Control and Prevention (CDC) Wide-ranging online data for epidemiologic research mortality data. Understanding these trends is essential to identifying high-risk populations and informing targeted screening and early intervention strategies. The findings can guide integrated smoking cessation and liver disease management efforts to reduce preventable mortality in vulnerable groups. This descriptive study utilized CDC WONDER data for adults aged 25 years and older. Mortality cases were identified using the International Classification of Diseases, tenth revision codes K72 (Liver failure) and F17 (MBD due to tobacco use). Age-adjusted mortality rates (AAMRs) per 100,000 were computed and stratified by gender, race/ethnicity, census region, Urban-Rural status, and state. Joinpoint regression was applied to estimate annual percent changes with 95% confidence intervals, and statistical significance was defined as *P* < .05. A total of 46,227 deaths were analyzed, with most occurring in medical facilities (61.7%), followed by homes and nursing facilities. The overall AAMR rose from 0.074 in 1999 to 0.94 in 2020 and then slightly declined to 0.78 by 2023. Both females and males showed similar upward trends, with males having higher AAMRs. Non-Hispanic Whites experienced the largest long-term increase, while Hispanic and Black populations showed recent declines. Mortality increased more sharply in rural areas as compared to urban areas, and regionally, the Midwest and South had sharp rises. State-level rates were highest in North Dakota, Wyoming, and South Dakota and lowest in California, Virginia, and Massachusetts, highlighting significant geographic disparities in liver failure mortality among adults with MBDs due to tobacco use. Deaths due to liver failure in adults with MBDs due to tobacco use in the United States increased substantially in the last 20 years, with significant demographic and regional inequalities. These results indicate the necessity of targeted prevention, smoking cessation programs, and equitable healthcare policies in order to decrease the mortality rate.

## Graphical abstract

**Figure:**
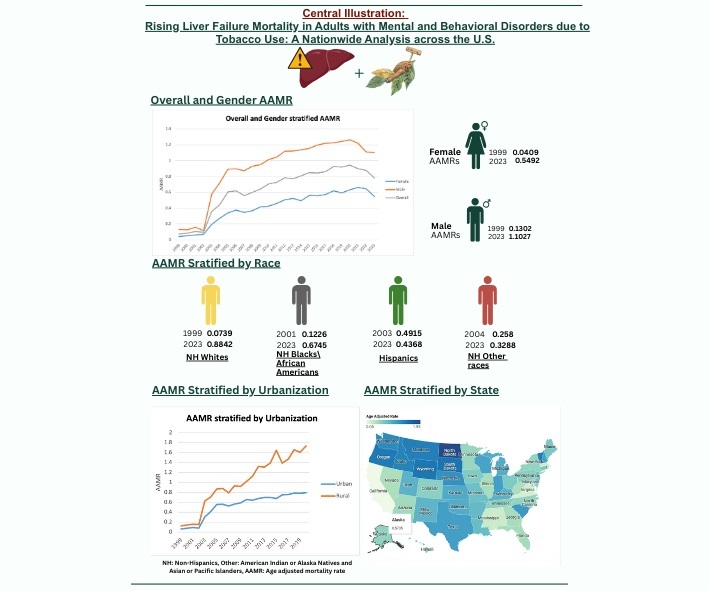

## 1. Introduction

Liver failure has emerged as the leading contributor to morbidity and mortality worldwide by triggering hepatic and extrahepatic organ failure.^[1]^ Liver failure is a grave condition characterized by acute decompensation of liver function. The loss of liver function is attributed to activation of the cellular immune response, systemic inflammation, and multiple organ dysfunction.^[2]^ Liver failure can be acute or chronic, depending on preexisting liver injury. In acute liver failure, any injury to the liver that cannot be compensated for leads to hepatocyte demise and eventually impairs liver function.^[3]^ Chronic liver failure is observed in patients suffering from chronic liver disease. It is acute decompensation precipitated by chronic liver disease. The etiology of liver failure is multifactorial. The predominant causes include hepatitis B, hepatitis A, and hepatitis E infections, alcohol-induced hepatitis, and bacterial infections. Drug-induced liver injury also contributes significantly to liver failure.^[4]^ Acetaminophen overdose is also known to be a principal etiological factor in liver injury.^[5]^ The global prevalence of liver failure is slightly higher than one per million. In the United States, 39% of the cases of liver failure are due to acetaminophen overdose.^[6]^

There is a significant correlation between adults with mental and behavioral disorders (MBDs) due to tobacco use and liver failure. Smoking and tobacco consumption are known to exacerbate chronic liver diseases, which may ultimately result in deterioration of liver function and liver failure.^[7]^ This contributes to the overall high mortality in this group. While previous studies have analyzed mortality trends in patients with liver failure, none have focused on its correlation in adults with MBDs due to tobacco use. To bridge this literature gap, our study has sourced data from the Centers for Disease Control (CDC), Wide-ranging online data for epidemiologic research (WONDER) to assess mortality in the United States from 1999 to 2023, categorized by year, gender, urbanization, state, census, region, and race, to identify populations at high risk.

## 2. Methods

The data were acquired through multiple cause of death files of the Centers for Disease Control and Prevention CDC Wide-ranging Online Data for Epidemiologic Research (WONDER), from 1999 to 2023.^[8]^ CDC WONDER is an electronic information and communications network dealing in the field of public health and giving access to statistical research data published by the CDC. Multiple causes of death files were used to extract deaths related to liver failure and MBDs due to tobacco use in adults aged ≥25 years. These diseases were identified using the International Classification of Diseases, tenth revision (ICD-10). Liver failure was defined using code K72 (hepatic failure, not elsewhere classified). Tobacco smoking was defined using code F17 (MBD due to use of tobacco). Other literature published in the past used the same ICD-10 codes.^[9–12]^ The data for only those death files were extracted in which both hepatic failure and MBDs due to tobacco use were listed as a cause of death anywhere on the death certificate.

The research utilized only de-identified and publicly accessible data and thus did not require review by the Institutional Review Board. The observational study was documented in accordance with the Strengthening the Reporting of Observational Studies in Epidemiology guidelines.^[13]^

### 2.1. Data extraction

Data were obtained for the whole adult population, disaggregated in relation to sex, race/ethnicity, 10-year age bracket (25–34 through ≥85), and the United States urbanization (metropolitan/nonmetropolitan), the state the person lived in, and where the person died. Race and ethnicity started to be documented based on the information reported by the funeral directors and by the next of kin. The race/ ethnic composition was categorized into Hispanic (Latino) and non-Hispanic (NH), where the latter was divided into NH White, NH Black, and a combined group (American Indians and Asians or Pacific Islanders) as NH Other. This combination of races classified as NH Other subgroup was used to avoid unreliable estimates due to low death counts. According to the United States Census Bureau, the United States had 4 regions, namely the Northeast, Midwest, South, and West. The level of urbanization was operationalized with the help of the Urban-Rural Classification Scheme of the National Center of Health Statistics, 2013.^[14]^ Counties that had a population of 50,000 or higher were metropolitan; the others were non-metropolitan.

The Northeast Census region data in the year 2000 was not reliable, hence the joinpoint analysis of the Northeast Census region began in 2001. In the same way, the joinpoint analysis was conducted over 2004 to 2023, in all races except NH Whites, because of the unreliable values in earlier years. This is in accordance with CDC guidelines.

We did not use annual data cells with fewer than 10 deaths (as recommended by CDC WONDER) because of unstable estimates in the data.^[15]^ This suppression limited the analysis of long-term trends in some subgroups, especially younger age groups and populations of smaller racial/ethnic groups. However, the suppressed data is included in the totals collected by CDC WONDER. Thus, they were not left out in the overall analysis.^[16]^

### 2.2. Statistical analysis

The CDC WONDER provided annual mortality rates and population data. The Direct method of calculating age-adjusted mortality rate (AAMRs) was applied, and AAMRs were standardized to the 2000 United States. standard population.^[17]^ The AAMRs were computed per 100,000 of the population, and the 95 percent confidence interval (CI) was determined according to the Poisson distribution assumption.^[18]^ AAMRs were used to measure race/ethnicity, gender, census region, state, age group, and urban-rural classification mortality trends.

Temporal trends were also evaluated with the help of the joinpoint regression relying on the log-linear models that yielded estimates of the annual percent change (APC) values in individual segments and the average annual percent change (AAPC) value of the entire study period. The analysis was carried out with the National Cancer Institute’s Joinpoint Regression software (version 5.4.0).^[19]^ The model started with one linear segment, and the addition of joinpoints was done in a statistically significant manner by the Monte Carlo permutation test.^[20]^

The empirically chosen best model fit determined the number and location of joinpoints. A *P*-value <.05 was considered significant. Separate subgroup-specific models were specified based on sex, race/ethnicity, age group, region of the country, urbanization level, and the state of residence. Each segment was reported with APCs and respective 95% CIs. APC/AAPCs/Tau CIs were calculated by using a parametric method.

## 3. Results

A total of 46,227 liver failure-related deaths among adults with MBDs due to tobacco use occurred between 1999 and 2023. Most of them took place at medical settings, accounting for 61.7% (n = 28, 545), followed by nursing homes (7.9%, n = 3668) and hospice facilities (6.7% n = 3075), respectively. (Supplementary Table 1)

### 3.1. Overall and gender stratified trends

The overall AAMR increased from 0.073 in 1999 to 0.78 in 2023 (AAPC = 13.98*; 95% CI: 11.10 to 16.94), peaking at 0.94 in 2020.

In females, the AAMR first rose sharply from 0.04 in 1999 to 0.34 in 2005 (APC = 46.91*; 95% CI: 34.79 to 60.12), followed by a slight but continuous increase to 0.66 in 2021 (APC = 4.10*; 95% CI: 3.25 to 4.96). For the entire study period, there was an AAPC of 12.23* in females (95% CI: 9.53–14.99).

**Figure 1.**
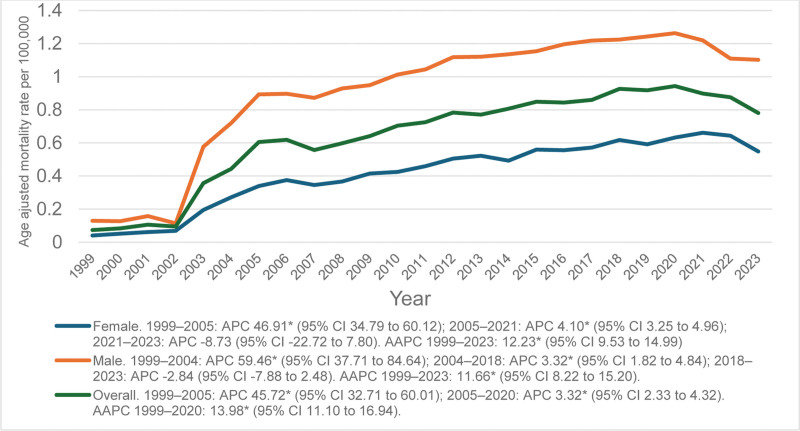
Trends in age-adjusted mortality rates due to liver failure among adults with MBDs due to tobacco use in the United States from 1999 to 2023, stratified by gender. The “*” sign indicates that the trend is statistically significant at alpha = 0.05. APC = annual percent change, CI = confidence interval, MBDs = mental and behavioral disorders.

In males, on the other hand, AAMR increased sharply from 0.13 in 1999 to 0.72 in 2004 (APC = 59.46*; 95% CI: 37.71–84.64). The AAMR continued to rise and reached 1.22 in the year 2018 (APC = 3.32*; 95% CI: 1.82–4.84). Then there was a nonsignificant decline in AAMR to 1.1 in 2023 (APC = -2.84; 95% CI: −7.88 to 2.48). Across the full study period, males had an AAPC of 11.66* (95% CI: 8.22 to 15.20). (Fig 1; Supplementary Table 2)

### 3.2. Race stratified trends

Among NH Whites, mortality rates increased steeply from 0.09 in 1999 to 0.47 in 2004 (APC = 65.04*; 95% CI: 43.90 to 89.29), followed by a slower but steady rise to 1.05 in 2018 (APC = 4.03*; 95% CI: 2.67 to 5.41). Rates then remained stable through 2023 (APC = −1.83; 95% CI:−6.41 to 2.98). Overall, the period from 1999 to 2023 demonstrated a significant increase among Whites (AAPC = 13.16*; 95% CI: 9.92 to 16.49).

Among Hispanic or Latino populations, rates increased modestly from 0.41 in 2004 to 0.56 in 2019 (APC = 2.21*; 95% CI: 1.21 to 3.21), followed by a decline to 0.44 in 2023 (APC = −5.64*; 95% CI:−10.89 to −0.08). The overall trend from 2004 to 2023 was not statistically significant (AAPC = 0.50; 95% CI:−0.81 to 1.84).

For NH Black or African American individuals, mortality rates rose from 0.42 in 2004 to 0.77 in 2017 (APC = 4.04*; 95% CI: 2.70 to 5.39), followed by stabilization through 2023 (APC = −1.14; 95% CI:−4.35 to 2.17). The overall trend from 2004 to 2023 showed a significant increase (AAPC = 2.37*; 95% CI: 1.09 to 3.66).

Among the NH Other group, mortality rates increased gradually from 0.26 in 2004 to 0.33 in 2023 (AAPC = 2.09*; 95% CI: 0.52 to 3.69).

**Figure 2.**
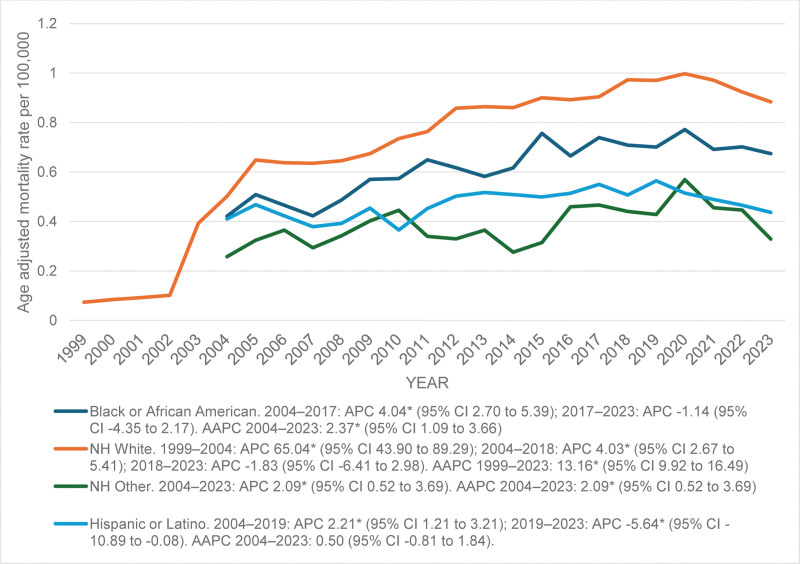
Trends in age-adjusted mortality rates due to liver failure among adults with MBDs due to tobacco use in the United States from 1999 to 2023, stratified by race. The “*” sign indicates that the trend is statistically significant at alpha = 0.05. APC = annual percent change, CI = confidence interval, MBDs = mental and behavioral disorders.

Overall, NH Whites exhibited the steepest increase in mortality, followed by slight increases in NH Black or African Americans, Hispanics, and NH Other, respectively. (Fig 2; Supplementary Table 3)

### 3.3. Urban/Rural stratified trends

Among urban (metropolitan) populations, the AAMR increased markedly from 0.06 in 1999 to 0.56 in 2005 (APC = 48.17*, 95% CI: 35.27 to 62.31), followed by a moderate rise to 0.79 in 2020 (APC = 2.60*, 95% CI: 1.67–3.54). In the entire study period, urban areas demonstrated an overall AAPC of 13.96* (95% CI: 11.15 to 16.84).

**Figure 3.**
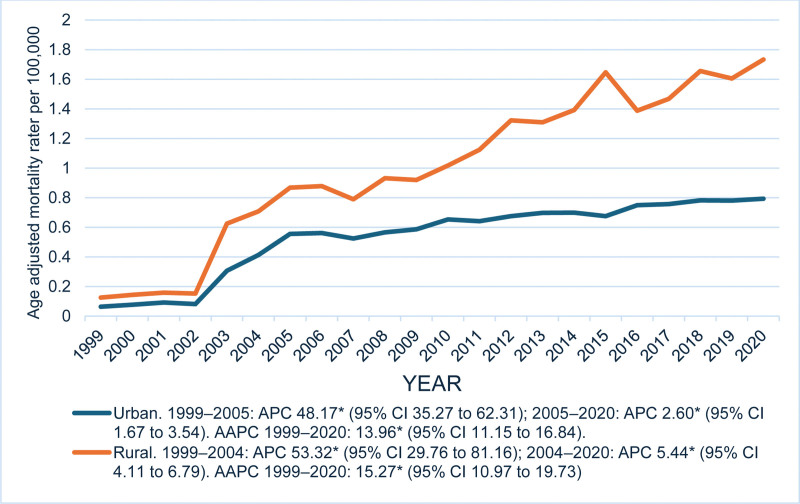
Trends in age-adjusted mortality rates due to liver failure among adults with MBDs due to tobacco use in the United States from 1999 to 2023, stratified by urban/metro and rural/nonmetro areas. The ‘*’ sign indicates that the trend is statistically significant. APC = annual percent change, CI = confidence interval, MBDs = mental and behavioral disorders.

Among rural (non-metropolitan) populations, the AAMR rose steeply from 0.13 in 1999 to 0.70 in 2004 (APC = 53.32*, 95% CI: 29.76 to 81.16), followed by a continued increase to 1.73 in 2020 (APC: 5.44*, 95% CI: 4.11 to 6.79). Across all the years from 1999 to 2020, rural areas exhibited an overall AAPC of 15.27* (95% CI = 10.97 to 19.73). (Fig 3; Supplementary Table 4)

### 3.4. Census region stratified trends

In the Midwest region, the AAMR rose steeply from 0.06 in 1999 to 0.50 in 2004 (APC: 56.94*, 95% CI: 35.23 to 82.13), followed by a continued rise to 0.96 in year 2010 (APC = 16.73*, 95% CI: 9.97 to 23.89). Then the trend further increased slightly to 1.27 in 2021 (APC = 2.11*, 95% CI: 0.62 to 3.61) before declining to 1.01 in 2023. The Midwest had an overall AAPC of 14.21* (95% CI: 10.25 to 18.31).

In the South, the AAMR increased sharply from 0.11 in 1999 to 0.72 in 2004 (APC = 49.20*, 95% CI: 23.43–80.34), followed by a gradual rise to 0.89 in 2023 (APC = 3.23*, 95% CI: 2.10 to 4.37). The South exhibited an overall AAPC of 11.46* (95% CI: 7.31 to 15.78).

In the West, the AAMR increased markedly from 0.09 in 1999 to 0.48 in 2004 (APC = 50.51*, 95% CI: 30.31 to 73.85), followed by a slower but consistent rise to 0.57 in 2023 (APC = 1.98*, 95% CI: 1.14 to 2.84). The West demonstrated an overall AAPC of 10.60* (95% CI: 7.45 to 13.84).

**Figure 4.**
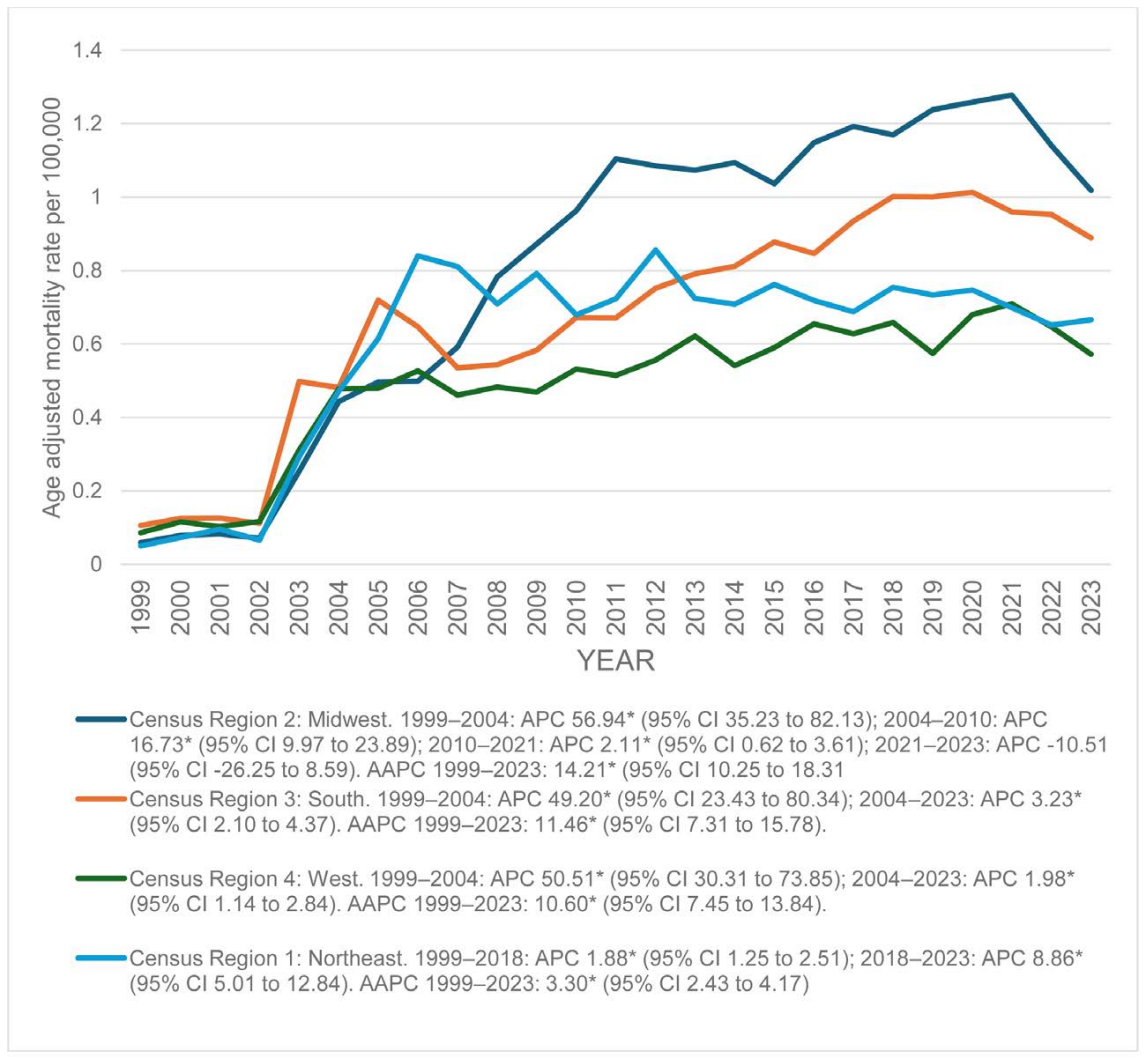
Trends in age-adjusted mortality rates due to liver failure among adults with MBDs due to tobacco use in the United States from 1999 to 2023, stratified by census region. The “*” sign indicates that the trend is statistically significant at alpha = 0.05. AAPC = average annual percent change, APC = annual percent change, CI = confidence interval, MBDs = mental and behavioral disorders.

In the Northeast region, the AAMR increased from 0.05 in 1999 to a peak of 0.84 in 2006 (APC = 1.88*, 95% CI: 1.25 to 2.51), after that there was a modest decline in subsequent years to 0.67 by 2023. In the entire study period, the Northeast region had an AAPC 3.30* (95% CI: 2.43 to 4.17). (Fig 4; Supplementary Table 5)

### 3.5. State stratified trends

**Figure 5.**
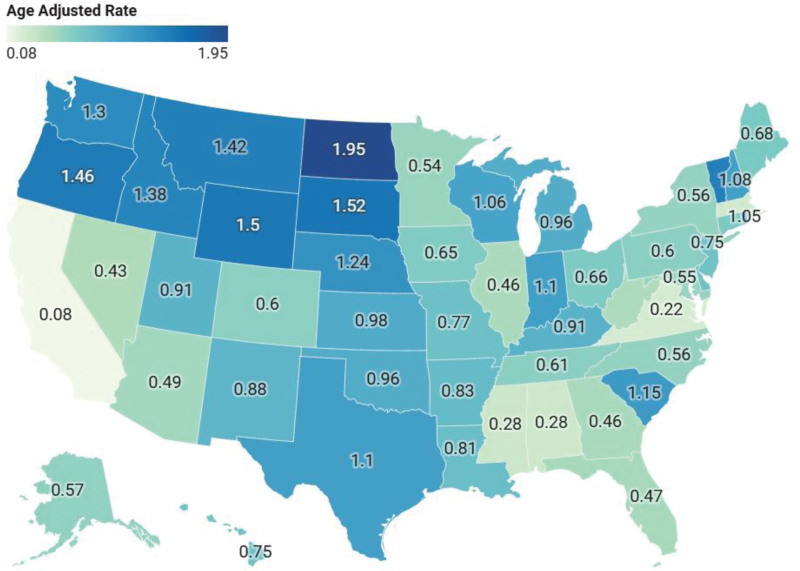
State-wise distribution of age-adjusted mortality rates due to liver failure among adults with MBDs due to tobacco use in the United States from 1999 to 2020. Darker shades represent higher mortality rates, while lighter shades indicate lower rates.

The highest AAMRs were observed in North Dakota (1.95), Wyoming (1.50), South Dakota (1.52), and Oregon (1.46), indicating substantially elevated rates in these states. On the other hand, the lowest AAMRs were recorded in Virginia (0.22), California (0.08), Massachusetts (0.24), and Alabama (0.28), reflecting relatively lower mortality burdens. (Fig 5; Supplementary Table 6)

## 4. Discussion

In this study, we analyzed the mortality rates of liver failure and its association with MBDs due to tobacco use from 1999 to 2023. Understanding these trends is critical for informing public health interventions and incorporating tobacco cessation strategies into liver failure management, with the goal of reducing mortality rates.

From 1999 to 2023, AAMR increased overall, peaking around 2020. Females and males showed similar upward trends. NH White and Black individuals had notable increases, with smaller rises among Hispanics and other races. Rural populations consistently had higher mortality than urban areas. Regionally, the Midwest showed the largest increase, followed by the South, Northeast, and West. At the state level, North Dakota had the highest AAMR (1.95), while Virginia had the lowest (0.22).

Studies show that liver diseases caused over a million deaths in 2010.^[21]^ A significant correlation is present between liver failure and smoking, the effects of tobacco on hepatic function, in addition to its cardiovascular and pulmonary effects. Evidence suggests that smoking is a major risk factor for liver fibrosis and cancer. Tobacco constituents, such as nicotine, promote systemic inflammation and oxidative stress, resulting in hepatocellular injury that may progress to fibrosis. Tobacco smoke contains free radicals and reactive oxygen species, which induce oxidative damage in hepatocytes, causing lipid peroxidation and DNA damage, ultimately contributing to the development of hepatic fibrosis over time. Furthermore, nicotine causes vasoconstriction, leading to hypoxia.^[7]^ In people with liver disease, smoking may exacerbate the situation and increase the likelihood of hepatic decompensation. Studies have demonstrated elevated liver enzyme levels and increased cirrhosis prevalence among smokers. This study further highlights a rising incidence of liver failure-related mortality associated with smoking, which has been linked to over 175 million deaths globally between 1990 and 2020.^[22]^ Even secondhand smoke has negative effects on the liver and is associated with nonalcoholic fatty liver in children.^[23]^

The difference in AAMR in males and females indicates the lifestyle differences and disparities in health-seeking patterns, with males having higher mortality as compared to females due to liver failure.^[9]^ Men are more involved in smoking and alcohol consumption than women. The steep initial rise among females (1999–2005) is owed to lifestyle shifts (like alcohol intake and obesity), evolving social norms, and delayed diagnosis.^[24]^ The stabilization of mortality rates after 2015 may be attributed to improved healthcare interventions, including greater access to antiviral therapies. Biologically, estrogen exerts hepatoprotective effects by reducing hepatic lipid peroxidation, a key driver of fibrosis, whereas androgens can exacerbate liver injury.^[25]^ This persistent male predominance highlights the need for targeted anti-smoking interventions.

This study demonstrated consistently higher AAMR in rural areas, likely due to higher smoking prevalence, low cessation rates, limited health awareness, reduced access to healthcare, delayed diagnosis, and suboptimal management strategies.^[26]^ While the urban population showed overall lower mortality rates as compared to rural areas, the initial rise till 2005 may be attributed to urban lifestyle changes leading to increased exposure to environmental toxins and smoking. Overall, the urban population benefited from public health measures and better smoking control and cessation strategies that contributed to the slow rise in mortality observed after 2010.^[27]^

This study reveals that NH White individuals exhibited the highest AAMR, consistent with the prevalent smoking, tobacco, and alcohol usage. Smoking combined with alcohol consumption worsens the situation, common to this race group.^[28]^ And the plateau after 2015 indicates successful smoking cessation strategies. Among Hispanic individuals, mortality trends showed a decline after 2019, attributable to lower smoking prevalence in these areas.

NH Black or African American populations exhibited a rise till 2017 and then a slight decline. Although smoking prevalence in these groups is lower than in Whites, the usage of menthol cigarettes and smoking for longer durations is due to smoking dependence and lower cessation rates.^[29]^ The gradual increase in Hispanics and NH Other racial groups may be attributed to poor lifestyle and eating habits.

The Midwest and South exhibited the highest increase in mortality, while the Northeast and West showed comparatively lower rates. These patterns are consistent with prior evidence indicating higher smoking prevalence in the Midwest and South, while the decline observed in the Northeast and West likely reflects effective tobacco control policies and improved healthcare access.

The elevated mortality rates observed in North Dakota, South Dakota, Wyoming, and Oregon may be explained by high smoking prevalence combined with limited access to healthcare services. States such as California, Virginia, and Massachusetts, which exhibit the lowest AAMRs, may indicate good smoking cessation policies and access to healthcare.

These findings suggest an increase in the smoking-related liver failure across demographic and geographic subgroups from 1999 to 2023. This rise reflects changes in lifestyle, socioeconomic differences, and healthcare access, and the recent stabilization in trends in some subgroups may suggest smoking cessation. However, the overall increase highlights the need for prevention strategies. Strengthening smoking cessation measures, early screening for liver failure and better access to healthcare could reduce smoking-associated liver disease mortalities.

### 4.1. Limitations

There are several limitations in this study. First, the use of death certificate data on the CDC WONDER database will provide a chance of misclassification or underreporting of liver failure (ICD-10 codes K72) and MBDs due to tobacco use (ICD-10 code F17) as underlying or contributing causes of death. Second, the database is not that detailed clinically, such as disease stage, history of treatment and smoking, comorbidities, and socioeconomic determinants of health, limiting the level of interpretation. Third, reporting of race and ethnicity on death certificates could be misclassified, especially among American Indian/ Alaska Native and Asian or Pacific Islander, thus biasing the subgroup analysis. Fourth, the inconsistency in the coding and classification could have been brought about by the variation in death certificate reporting practices across states and times. Fifth, the Coronavirus disease 2019 pandemic could have indirectly impacted care continuity and healthcare-seeking behavior and could have influenced mortality trends in 2020 to 2021. Lastly, since it is an ecological, mortality-based study, no association can be construed as causal.

## 5. Conclusion

Liver failure mortality among adults with MBDs due to tobacco use has shown a consistent rise over the past 2 decades, with the highest rates observed in rural and southern states, reflecting notable regional disparities. Public health policies must prioritize stronger tobacco regulation and taxation while expanding access to smoking cessation and liver screening programs. Additionally, nationwide education campaigns promoting early intervention and lifestyle modification are essential. Coordinated national efforts are urgently needed to address these preventable deaths and reduce the growing burden of liver failure linked to tobacco use. Future studies should explore underlying mechanisms and healthcare access barriers contributing to these disparities to inform more precise, population-specific prevention and treatment strategies.

## Author contributions

**Conceptualization:** Sarim Hassan Shahab, Syeda Malika Naqvi.

**Project administration:** Sarim Hassan Shahab.

**Resources:** Sarim Hassan Shahab, Alisha Matloob.

**Software:** Sarim Hassan Shahab.

**Visualization:** Sarim Hassan Shahab, Zuha Maryam, Syeda Malika Naqvi, Hadia Ghazala Masood.

**Validation:** Zuha Maryam, Syeda Malika Naqvi, Alisha Matloob, Hadia Ghazala Masood.

**Data curation:** Syeda Malika Naqvi, Muhammad Ahmed.

**Formal analysis:** Muhammad Ahmed.

**Methodology:** Muhammad Ahmed.

**Supervision:** Hadia Ghazala Masood, Muddassir Khalid.

**Writing – original draft:** Sarim Hassan Shahab, Zuha Maryam, Syeda Malika Naqvi, Muhammad Ahmed, Alisha Matloob, Hadia Ghazala Masood, Esha Umair, Muhammad Talha, Ubaid Ur Rehman.

**Writing – review & editing:** Sarim Hassan Shahab, Zuha Maryam, Syeda Malika Naqvi, Muhammad Ahmed, Alisha Matloob, Hadia Ghazala Masood, Esha Umair, Muddassir Khalid, Muhammad Talha, Ubaid Ur Rehman.












