# Supplementary material for: Trends and disparities in liver failure-related mortality in adults with mental and behavioral disorders due to tobacco use: A retrospective analysis
Source: Medicine (Baltimore). 2026 May 15;105(20):e48719. doi: 10.1097/MD.0000000000048719 (PMC13183028; doi:10.1097/MD.0000000000048719)
Supplement: Supplementary file 1 [file medi-105-e48719-s001.docx]

Supplementary Table 1. Place of death data table due to liver failure among adults with mental and behavioral disorders due to tobacco use

| **Place of Death** | **Deaths** | **% of Total Deaths** |
| --- | --- | --- |
| Medical Facility | 28545 | 61.74% |
| Decedent's home | 9878 | 21.37% |
| Hospice facility | 3075 | 6.65% |
| Nursing home/long term care | 3668 | 7.93% |
| Other | 1038 | 2.24% |
| Place of death Unknown | 23 | 0.05% |
| Total | 46227 | 100% |
