# Supplementary material for: Trends and disparities in liver failure-related mortality in adults with mental and behavioral disorders due to tobacco use: A retrospective analysis
Source: Medicine (Baltimore). 2026 May 15;105(20):e48719. doi: 10.1097/MD.0000000000048719 (PMC13183028; doi:10.1097/MD.0000000000048719)
Supplement: Supplementary file 2 [file medi-105-e48719-s002.docx]

Supplementary Table 2. Gender stratified and overall mortality data due to liver failure among adults with mental and behavioral disorders due to tobacco use

| **Year** | **Sex** | **Age Adjusted Rate (95% CI)** | **Sex** | **Age Adjusted Rate (95% CI)** | **Overall** | **Age Adjusted Rate (95% CI)** |
| --- | --- | --- | --- | --- | --- | --- |
| 1999 | Female | 0.04 (0.03–0.06) | Male | 0.13 (0.10–0.16) | Overall (M+F) | 0.07 (0.06–0.09) |
| 2000 | Female | 0.05 (0.04–0.07) | Male | 0.13 (0.10–0.15) | Overall (M+F) | 0.08 (0.07–0.10) |
| 2001 | Female | 0.06 (0.05–0.08) | Male | 0.16 (0.13–0.19) | Overall (M+F) | 0.11 (0.09–0.12) |
| 2002 | Female | 0.07 (0.05–0.09) | Male | 0.11 (0.09–0.14) | Overall (M+F) | 0.09 (0.08–0.11) |
| 2003 | Female | 0.20 (0.17–0.22) | Male | 0.58 (0.53–0.63) | Overall (M+F) | 0.36 (0.33–0.38) |
| 2004 | Female | 0.27 (0.24–0.30) | Male | 0.72 (0.66–0.78) | Overall (M+F) | 0.44 (0.41–0.47) |
| 2005 | Female | 0.34 (0.31–0.37) | Male | 0.89 (0.83–0.95) | Overall (M+F) | 0.61 (0.57–0.64) |
| 2006 | Female | 0.38 (0.34–0.41) | Male | 0.90 (0.84–0.96) | Overall (M+F) | 0.62 (0.58–0.65) |
| 2007 | Female | 0.35 (0.31–0.38) | Male | 0.87 (0.81–0.93) | Overall (M+F) | 0.56 (0.53–0.59) |
| 2008 | Female | 0.37 (0.33–0.40) | Male | 0.93 (0.87–0.99) | Overall (M+F) | 0.60 (0.57–0.63) |
| 2009 | Female | 0.42 (0.38–0.45) | Male | 0.95 (0.89–1.01) | Overall (M+F) | 0.64 (0.61–0.67) |
| 2010 | Female | 0.43 (0.39–0.46) | Male | 1.01 (0.95–1.08) | Overall (M+F) | 0.71 (0.67–0.74) |
| 2011 | Female | 0.46 (0.42–0.50) | Male | 1.04 (0.98–1.11) | Overall (M+F) | 0.73 (0.69–0.76) |
| 2012 | Female | 0.50 (0.46–0.55) | Male | 1.12 (1.06–1.18) | Overall (M+F) | 0.78 (0.75–0.82) |
| 2013 | Female | 0.52 (0.48–0.56) | Male | 1.12 (1.06–1.18) | Overall (M+F) | 0.77 (0.74–0.81) |
| 2014 | Female | 0.49 (0.45–0.53) | Male | 1.14 (1.07–1.20) | Overall (M+F) | 0.81 (0.77–0.84) |
| 2015 | Female | 0.56 (0.52–0.60) | Male | 1.15 (1.09–1.22) | Overall (M+F) | 0.85 (0.81–0.89) |
| 2016 | Female | 0.56 (0.51–0.60) | Male | 1.20 (1.13–1.26) | Overall (M+F) | 0.84 (0.81–0.88) |
| 2017 | Female | 0.57 (0.53–0.61) | Male | 1.22 (1.16–1.28) | Overall (M+F) | 0.86 (0.82–0.90) |
| 2018 | Female | 0.62 (0.57–0.66) | Male | 1.22 (1.16–1.29) | Overall (M+F) | 0.93 (0.89–0.96) |
| 2019 | Female | 0.59 (0.55–0.63) | Male | 1.24 (1.18–1.31) | Overall (M+F) | 0.92 (0.88–0.96) |
| 2020 | Female | 0.63 (0.59–0.68) | Male | 1.26 (1.20–1.33) | Overall (M+F) | 0.94 (0.91–0.98) |
| 2021 | Female | 0.66 (0.62–0.71) | Male | 1.22 (1.16–1.28) | Overall (M+F) | 0.90 (0.86–0.93) |
| 2022 | Female | 0.64 (0.60–0.69) | Male | 1.11 (1.05–1.17) | Overall (M+F) | 0.88 (0.84–0.91) |
| 2023 | Female | 0.55 (0.51–0.59) | Male | 1.10 (1.04–1.16) | Overall (M+F) | 0.78 (0.75–0.81) |
