# Supplementary material for: Trends and disparities in liver failure-related mortality in adults with mental and behavioral disorders due to tobacco use: A retrospective analysis
Source: Medicine (Baltimore). 2026 May 15;105(20):e48719. doi: 10.1097/MD.0000000000048719 (PMC13183028; doi:10.1097/MD.0000000000048719)
Supplement: Supplementary file 3 [file medi-105-e48719-s003.docx]

Supplementary Table 3. Race stratified mortality data due to liver failure among adults with mental and behavioral disorders due to tobacco use

| **Year** | **Race** | **Age-Adjusted Rate (95% CI)** | **Year2** | **Race3** | **Age-Adjusted Rate (95% CI)4** |
| --- | --- | --- | --- | --- | --- |
| 1999 | Hispanic or Latino | #N/A | 1999 | Black or African American | #N/A |
| 2000 | Hispanic or Latino | #N/A | 2000 | Black or African American | #N/A |
| 2001 | Hispanic or Latino | #N/A | 2001 | Black or African American | #N/A |
| 2002 | Hispanic or Latino | #N/A | 2002 | Black or African American | #N/A |
| 2003 | Hispanic or Latino | #N/A | 2003 | Black or African American | #N/A |
| 2004 | Hispanic or Latino | 0.41 (0.31–0.54) | 2004 | Black or African American | 0.42 (0.34–0.52) |
| 2005 | Hispanic or Latino | 0.47 (0.37–0.59) | 2005 | Black or African American | 0.51 (0.41–0.61) |
| 2006 | Hispanic or Latino | 0.42 (0.33–0.54) | 2006 | Black or African American | 0.47 (0.37–0.56) |
| 2007 | Hispanic or Latino | 0.38 (0.29–0.49) | 2007 | Black or African American | 0.42 (0.34–0.52) |
| 2008 | Hispanic or Latino | 0.39 (0.31–0.49) | 2008 | Black or African American | 0.49 (0.39–0.58) |
| 2009 | Hispanic or Latino | 0.45 (0.36–0.57) | 2009 | Black or African American | 0.57 (0.47–0.67) |
| 2010 | Hispanic or Latino | 0.37 (0.28–0.46) | 2010 | Black or African American | 0.57 (0.48–0.67) |
| 2011 | Hispanic or Latino | 0.45 (0.36–0.56) | 2011 | Black or African American | 0.65 (0.55–0.75) |
| 2012 | Hispanic or Latino | 0.50 (0.40–0.60) | 2012 | Black or African American | 0.62 (0.52–0.72) |
| 2013 | Hispanic or Latino | 0.52 (0.42–0.62) | 2013 | Black or African American | 0.58 (0.49–0.68) |
| 2014 | Hispanic or Latino | 0.51 (0.42–0.60) | 2014 | Black or African American | 0.62 (0.52–0.71) |
| 2015 | Hispanic or Latino | 0.50 (0.41–0.59) | 2015 | Black or African American | 0.76 (0.65–0.86) |
| 2016 | Hispanic or Latino | 0.51 (0.42–0.61) | 2016 | Black or African American | 0.67 (0.57–0.76) |
| 2017 | Hispanic or Latino | 0.55 (0.46–0.64) | 2017 | Black or African American | 0.74 (0.64–0.84) |
| 2018 | Hispanic or Latino | 0.51 (0.42–0.59) | 2018 | Black or African American | 0.71 (0.61–0.81) |
| 2019 | Hispanic or Latino | 0.56 (0.47–0.66) | 2019 | Black or African American | 0.70 (0.61–0.79) |
| 2020 | Hispanic or Latino | 0.52 (0.43–0.60) | 2020 | Black or African American | 0.77 (0.67–0.87) |
| 2021 | Hispanic or Latino | 0.49 (0.41–0.57) | 2021 | Black or African American | 0.69 (0.60–0.79) |
| 2022 | Hispanic or Latino | 0.47 (0.39–0.54) | 2022 | Black or African American | 0.70 (0.61–0.80) |
| 2023 | Hispanic or Latino | 0.44 (0.36–0.51) | 2023 | Black or African American | 0.67 (0.58–0.77) |
| **Year** | **Race** | **Age-Adjusted Rate (95% CI)** | **Year** | **Race** | **Age-Adjusted Rate (95% CI)** |
| 1999 | NH Other | #N/A | 1999 | White | 0.07 (0.06–0.09) |
| 2000 | NH Other | #N/A | 2000 | White | 0.08 (0.07–0.10) |
| 2001 | NH Other | #N/A | 2001 | White | 0.09 (0.08–0.11) |
| 2002 | NH Other | #N/A | 2002 | White | 0.10 (0.09–0.12) |
| 2003 | NH Other | #N/A | 2003 | White | 0.39 (0.36–0.42) |
| 2004 | NH Other | 0.26 (0.16–0.39) | 2004 | White | 0.50 (0.47–0.54) |
| 2005 | NH Other | 0.32 (0.22–0.47) | 2005 | White | 0.65 (0.61–0.69) |
| 2006 | NH Other | 0.37 (0.25–0.51) | 2006 | White | 0.64 (0.60–0.68) |
| 2007 | NH Other | 0.29 (0.19–0.44) | 2007 | White | 0.64 (0.60–0.67) |
| 2008 | NH Other | 0.34 (0.23–0.48) | 2008 | White | 0.65 (0.61–0.68) |
| 2009 | NH Other | 0.40 (0.29–0.54) | 2009 | White | 0.67 (0.64–0.71) |
| 2010 | NH Other | 0.45 (0.33–0.59) | 2010 | White | 0.74 (0.70–0.77) |
| 2011 | NH Other | 0.34 (0.25–0.45) | 2011 | White | 0.76 (0.72–0.80) |
| 2012 | NH Other | 0.33 (0.23–0.45) | 2012 | White | 0.86 (0.82–0.90) |
| 2013 | NH Other | 0.37 (0.27–0.49) | 2013 | White | 0.86 (0.82–0.91) |
| 2014 | NH Other | 0.28 (0.20–0.37) | 2014 | White | 0.86 (0.82–0.90) |
| 2015 | NH Other | 0.32 (0.23–0.42) | 2015 | White | 0.90 (0.86–0.94) |
| 2016 | NH Other | 0.46 (0.36–0.58) | 2016 | White | 0.89 (0.85–0.93) |
| 2017 | NH Other | 0.47 (0.37–0.59) | 2017 | White | 0.90 (0.86–0.95) |
| 2018 | NH Other | 0.44 (0.35–0.55) | 2018 | White | 0.97 (0.93–1.02) |
| 2019 | NH Other | 0.43 (0.34–0.54) | 2019 | White | 0.97 (0.93–1.01) |
| 2020 | NH Other | 0.57 (0.46–0.68) | 2020 | White | 1.00 (0.95–1.04) |
| 2021 | NH Other | 0.46 (0.36–0.57) | 2021 | White | 0.97 (0.93–1.01) |
| 2022 | NH Other | 0.45 (0.35–0.56) | 2022 | White | 0.92 (0.88–0.97) |
| 2023 | NH Other | 0.33 (0.25–0.43) | 2023 | White | 0.88 (0.84–0.92) |
