# Supplementary material for: Trends and disparities in liver failure-related mortality in adults with mental and behavioral disorders due to tobacco use: A retrospective analysis
Source: Medicine (Baltimore). 2026 May 15;105(20):e48719. doi: 10.1097/MD.0000000000048719 (PMC13183028; doi:10.1097/MD.0000000000048719)
Supplement: Supplementary file 4 [file medi-105-e48719-s004.docx]

Supplementary Table 4. Urban and rural wise mortality data due to liver failure among adults with mental and behavioral disorders due to tobacco use

| **Year** | **Area** | **Age adjusted rate (95% CI)** | **Year** | **Area** | **Age adjusted rate (95% CI)** |
| --- | --- | --- | --- | --- | --- |
| 1999 | Urban (Metropolitan) | 0.06 (0.05–0.08) | 1999 | Rural (Non-metropolitan) | 0.13 (0.09–0.17) |
| 2000 | Urban (Metropolitan) | 0.08 (0.06–0.09) | 2000 | Rural (Non-metropolitan) | 0.14 (0.11–0.19) |
| 2001 | Urban (Metropolitan) | 0.09 (0.08–0.11) | 2001 | Rural (Non-metropolitan) | 0.16 (0.12–0.21) |
| 2002 | Urban (Metropolitan) | 0.08 (0.07–0.10) | 2002 | Rural (Non-metropolitan) | 0.15 (0.11–0.20) |
| 2003 | Urban (Metropolitan) | 0.31 (0.28–0.33) | 2003 | Rural (Non-metropolitan) | 0.63 (0.54–0.71) |
| 2004 | Urban (Metropolitan) | 0.41 (0.38–0.45) | 2004 | Rural (Non-metropolitan) | 0.71 (0.62–0.80) |
| 2005 | Urban (Metropolitan) | 0.56 (0.52–0.59) | 2005 | Rural (Non-metropolitan) | 0.87 (0.77–0.97) |
| 2006 | Urban (Metropolitan) | 0.56 (0.53–0.60) | 2006 | Rural (Non-metropolitan) | 0.88 (0.78–0.98) |
| 2007 | Urban (Metropolitan) | 0.52 (0.49–0.56) | 2007 | Rural (Non-metropolitan) | 0.79 (0.70–0.88) |
| 2008 | Urban (Metropolitan) | 0.57 (0.53–0.60) | 2008 | Rural (Non-metropolitan) | 0.93 (0.83–1.03) |
| 2009 | Urban (Metropolitan) | 0.59 (0.55–0.62) | 2009 | Rural (Non-metropolitan) | 0.92 (0.82–1.02) |
| 2010 | Urban (Metropolitan) | 0.65 (0.62–0.69) | 2010 | Rural (Non-metropolitan) | 1.02 (0.91–1.12) |
| 2011 | Urban (Metropolitan) | 0.64 (0.61–0.68) | 2011 | Rural (Non-metropolitan) | 1.12 (1.02–1.23) |
| 2012 | Urban (Metropolitan) | 0.68 (0.64–0.71) | 2012 | Rural (Non-metropolitan) | 1.32 (1.21–1.44) |
| 2013 | Urban (Metropolitan) | 0.70 (0.66–0.73) | 2013 | Rural (Non-metropolitan) | 1.31 (1.19–1.43) |
| 2014 | Urban (Metropolitan) | 0.70 (0.66–0.74) | 2014 | Rural (Non-metropolitan) | 1.39 (1.27–1.51) |
| 2015 | Urban (Metropolitan) | 0.68 (0.64–0.71) | 2015 | Rural (Non-metropolitan) | 1.65 (1.51–1.78) |
| 2016 | Urban (Metropolitan) | 0.75 (0.71–0.79) | 2016 | Rural (Non-metropolitan) | 1.39 (1.27–1.51) |
| 2017 | Urban (Metropolitan) | 0.76 (0.72–0.79) | 2017 | Rural (Non-metropolitan) | 1.47 (1.35–1.59) |
| 2018 | Urban (Metropolitan) | 0.78 (0.75–0.82) | 2018 | Rural (Non-metropolitan) | 1.66 (1.52–1.79) |
| 2019 | Urban (Metropolitan) | 0.78 (0.74–0.82) | 2019 | Rural (Non-metropolitan) | 1.61 (1.48–1.73) |
| 2020 | Urban (Metropolitan) | 0.79 (0.76–0.83) | 2020 | Rural (Non-metropolitan) | 1.73 (1.60–1.87) |
