# Supplementary material for: Trends and disparities in liver failure-related mortality in adults with mental and behavioral disorders due to tobacco use: A retrospective analysis
Source: Medicine (Baltimore). 2026 May 15;105(20):e48719. doi: 10.1097/MD.0000000000048719 (PMC13183028; doi:10.1097/MD.0000000000048719)
Supplement: Supplementary file 6 [file medi-105-e48719-s006.docx]

Supplementary Table 6. Census region specified mortality data due to liver failure among adults with mental and behavioral disorders due to tobacco use

| **Census Region** | **Year** | **Age Adjusted Rate (95% CI)** | **Census Region2** | **Year3** | **Age Adjusted Rate (95% CI)4** |
| --- | --- | --- | --- | --- | --- |
| Census Region 1: Northeast | 1999 | 0.0498 (0.03–0.0778) | Census Region 2: Midwest | 1999 | 0.0591 (0.0393–0.0855) |
| Census Region 1: Northeast | 2000 | #N/A | Census Region 2: Midwest | 2000 | 0.0784 (0.0525–0.1126) |
| Census Region 1: Northeast | 2001 | 0.0956 (0.064–0.1373) | Census Region 2: Midwest | 2001 | 0.0821 (0.0558–0.1166) |
| Census Region 1: Northeast | 2002 | 0.0654 (0.0419–0.0972) | Census Region 2: Midwest | 2002 | 0.071 (0.0472–0.1027) |
| Census Region 1: Northeast | 2003 | 0.2925 (0.2385–0.3465) | Census Region 2: Midwest | 2003 | 0.2535 (0.204–0.303) |
| Census Region 1: Northeast | 2004 | 0.4714 (0.4028–0.5399) | Census Region 2: Midwest | 2004 | 0.4432 (0.3809–0.5056) |
| Census Region 1: Northeast | 2005 | 0.6142 (0.5361–0.6923) | Census Region 2: Midwest | 2005 | 0.4964 (0.4323–0.5605) |
| Census Region 1: Northeast | 2006 | 0.8396 (0.7479–0.9314) | Census Region 2: Midwest | 2006 | 0.4988 (0.4326–0.565) |
| Census Region 1: Northeast | 2007 | 0.8111 (0.7212–0.901) | Census Region 2: Midwest | 2007 | 0.5915 (0.5205–0.6624) |
| Census Region 1: Northeast | 2008 | 0.7093 (0.6282–0.7904) | Census Region 2: Midwest | 2008 | 0.782 (0.7032–0.8607) |
| Census Region 1: Northeast | 2009 | 0.7922 (0.705–0.8794) | Census Region 2: Midwest | 2009 | 0.8724 (0.7874–0.9575) |
| Census Region 1: Northeast | 2010 | 0.6787 (0.5996–0.7578) | Census Region 2: Midwest | 2010 | 0.9633 (0.8745–1.0521) |
| Census Region 1: Northeast | 2011 | 0.723 (0.6413–0.8048) | Census Region 2: Midwest | 2011 | 1.1037 (1.0102–1.1971) |
| Census Region 1: Northeast | 2012 | 0.8557 (0.7685–0.943) | Census Region 2: Midwest | 2012 | 1.085 (0.993–1.177) |
| Census Region 1: Northeast | 2013 | 0.7243 (0.6439–0.8047) | Census Region 2: Midwest | 2013 | 1.0732 (0.9829–1.1636) |
| Census Region 1: Northeast | 2014 | 0.7084 (0.6298–0.7871) | Census Region 2: Midwest | 2014 | 1.0937 (1.0038–1.1836) |
| Census Region 1: Northeast | 2015 | 0.7618 (0.6779–0.8457) | Census Region 2: Midwest | 2015 | 1.0365 (0.9491–1.1239) |
| Census Region 1: Northeast | 2016 | 0.7187 (0.6411–0.7963) | Census Region 2: Midwest | 2016 | 1.1484 (1.0559–1.241) |
| Census Region 1: Northeast | 2017 | 0.6877 (0.6133–0.762) | Census Region 2: Midwest | 2017 | 1.1923 (1.0977–1.2869) |
| Census Region 1: Northeast | 2018 | 0.7539 (0.675–0.8327) | Census Region 2: Midwest | 2018 | 1.1695 (1.0765–1.2624) |
| Census Region 1: Northeast | 2019 | 0.7332 (0.6539–0.8125) | Census Region 2: Midwest | 2019 | 1.2382 (1.1447–1.3317) |
| Census Region 1: Northeast | 2020 | 0.7465 (0.6674–0.8257) | Census Region 2: Midwest | 2020 | 1.2589 (1.1631–1.3547) |
| Census Region 1: Northeast | 2021 | 0.6987 (0.6236–0.7739) | Census Region 2: Midwest | 2021 | 1.2777 (1.1801–1.3754) |
| Census Region 1: Northeast | 2022 | 0.6518 (0.5782–0.7253) | Census Region 2: Midwest | 2022 | 1.1407 (1.0488–1.2326) |
| Census Region 1: Northeast | 2023 | 0.6655 (0.5924–0.7385) | Census Region 2: Midwest | 2023 | 1.018 (0.9318–1.1042) |
| Census Region 3: South | 1999 | 0.1058 (0.0806–0.1365) | Census Region 4: West | 1999 | 0.0858 (0.0555–0.1266) |
| Census Region 3: South | 2000 | 0.1245 (0.0978–0.1564) | Census Region 4: West | 2000 | 0.1152 (0.0826–0.1562) |
| Census Region 3: South | 2001 | 0.1254 (0.0997–0.1556) | Census Region 4: West | 2001 | 0.1026 (0.0726–0.1408) |
| Census Region 3: South | 2002 | 0.1111 (0.087–0.1396) | Census Region 4: West | 2002 | 0.1164 (0.0835–0.1579) |
| Census Region 3: South | 2003 | 0.4979 (0.4457–0.5501) | Census Region 4: West | 2003 | 0.3107 (0.2575–0.3638) |
| Census Region 3: South | 2004 | 0.4812 (0.4294–0.5329) | Census Region 4: West | 2004 | 0.478 (0.4107–0.5453) |
| Census Region 3: South | 2005 | 0.7196 (0.6581–0.7811) | Census Region 4: West | 2005 | 0.4793 (0.4148–0.5437) |
| Census Region 3: South | 2006 | 0.6469 (0.5886–0.7053) | Census Region 4: West | 2006 | 0.5273 (0.4586–0.596) |
| Census Region 3: South | 2007 | 0.5351 (0.4836–0.5866) | Census Region 4: West | 2007 | 0.4604 (0.3952–0.5256) |
| Census Region 3: South | 2008 | 0.5432 (0.4917–0.5946) | Census Region 4: West | 2008 | 0.4827 (0.4186–0.5468) |
| Census Region 3: South | 2009 | 0.5831 (0.5299–0.6363) | Census Region 4: West | 2009 | 0.4695 (0.4072–0.5317) |
| Census Region 3: South | 2010 | 0.672 (0.6157–0.7283) | Census Region 4: West | 2010 | 0.5319 (0.4679–0.5959) |
| Census Region 3: South | 2011 | 0.671 (0.6152–0.7268) | Census Region 4: West | 2011 | 0.5144 (0.452–0.5767) |
| Census Region 3: South | 2012 | 0.7515 (0.6923–0.8107) | Census Region 4: West | 2012 | 0.556 (0.491–0.621) |
| Census Region 3: South | 2013 | 0.7911 (0.7307–0.8515) | Census Region 4: West | 2013 | 0.6214 (0.5544–0.6885) |
| Census Region 3: South | 2014 | 0.8112 (0.7516–0.8707) | Census Region 4: West | 2014 | 0.5408 (0.4791–0.6025) |
| Census Region 3: South | 2015 | 0.8778 (0.8172–0.9384) | Census Region 4: West | 2015 | 0.5901 (0.5267–0.6535) |
| Census Region 3: South | 2016 | 0.8464 (0.7866–0.9063) | Census Region 4: West | 2016 | 0.6546 (0.5865–0.7226) |
| Census Region 3: South | 2017 | 0.9349 (0.8721–0.9977) | Census Region 4: West | 2017 | 0.6277 (0.5625–0.6929) |
| Census Region 3: South | 2018 | 1.0016 (0.9379–1.0654) | Census Region 4: West | 2018 | 0.6582 (0.5907–0.7258) |
| Census Region 3: South | 2019 | 1.0006 (0.9367–1.0644) | Census Region 4: West | 2019 | 0.5742 (0.5139–0.6346) |
| Census Region 3: South | 2020 | 1.0131 (0.9497–1.0766) | Census Region 4: West | 2020 | 0.68 (0.6129–0.7471) |
| Census Region 3: South | 2021 | 0.9599 (0.8985–1.0213) | Census Region 4: West | 2021 | 0.7092 (0.6397–0.7786) |
| Census Region 3: South | 2022 | 0.953 (0.891–1.0149) | Census Region 4: West | 2022 | 0.6464 (0.5827–0.71) |
| Census Region 3: South | 2023 | 0.8888 (0.831–0.9465) | Census Region 4: West | 2023 | 0.572 (0.5121–0.6318) |
